# Prognostic value of MMP9 activity level in resected stage I B lung adenocarcinoma

**DOI:** 10.1002/cam4.821

**Published:** 2016-07-25

**Authors:** Yongfeng Yu, Zhengping Ding, Hong Jian, Lan Shen, Lei Zhu, Shun Lu

**Affiliations:** ^1^ Shanghai Lung Cancer CenterShanghai Chest Hospital, Shanghai Jiao Tong University241 West Huaihai Road, Xuhui DistrictShanghai200030China; ^2^ Department of PathologyShanghai Chest Hospital, Shanghai Jiao Tong University241 West Huaihai Road, Xuhui DistrictShanghai200030China

**Keywords:** Activity, histological subtype, lung adenocarcinoma, MMP9

## Abstract

The clinical outcomes of patients with early‐stage non‐small cell lung cancer (NSCLC) have remained unsatisfactory after complete surgical resection. The objective of this study was to explore the prognostic value of matrix metalloproteinase 9 (MMP9) activity level in Chinese patients with stage I B lung adenocarcinoma. A sensitive and validated method was employed for determining the activity of MMP9 in human lung adenocarcinoma cells in vitro. Then, the association was examined between the level of MMP9 enzymatic activity and clinical outcomes. A total of 104 cases were stratified according to the IASLC/ATS/ERS classification scheme and activity of MMP9 was analyzed by SensoLyte® assay kit. The results showed that the MMP9 activity was the highest in solid predominant and micropapillary predominant subtypes, intermediate in acinar predominant and papillary predominant subtypes, and the lowest in lepidic predominant subtype. Multivariate analysis revealed that pathological subtype and activity of MMP9 were independent prognostic factors for disease‐free survival (DFS), respectively (*P = *0.005 and 0.029). Significant relationship existed between enzyme activity of MMP9 and prognosis. And the 30 months DFS of high‐ and low‐level MMP9 activity tumors was 44.2% and 84.1% (*P *<* *0.0001), respectively. High‐level MMP9 activity is correlated with aggressive tumor behaviors and poor clinical outcomes in early‐stage lung adenocarcinoma after complete resection.

## Introduction

Lung cancer is the leading cause of cancer‐related mortality worldwide 1. In recent decades, the most common histological type of non‐small cell lung carcinoma (NSCLC) has been lung adenocarcinoma, accounting for 70% of NSCLC and almost half of all lung carcinomas 2. With the widespread screening of computed tomography (CT), there has been a sharp increase in the detection and treatment of early‐stage lung cancer. However, the clinical outcomes have remained unsatisfactory for patients with stage I NSCLC after complete surgical resection. The 5‐year overall survival rates range from 55% to 77.6%. In other words, about one‐third of these patients with early‐stage disease died within 5 years. Some patients could survive while others died 3, 4, 5. It has been unknown as to why the outcomes varied so greatly. The current staging system failed to distinguish those patients at a higher risk of recurrence after surgical resection 6. Thus, it underscores an imperative need for better prognostic factors to identify patients at high risks of early recurrence after curative‐intent surgical resection.

In 2011, new terminology and diagnostic criteria for lung adenocarcinoma were proposed by the International Association for the Study of Lung Cancer/American Thoracic Society/European Respiratory Society (IASLC/ATS/ERS) 7. And the 2015 World Health Organization classification of lung adenocarcinoma was consistent with the above scheme in resection specimens 8. Previous reports showed that the former classification scheme had significant prognostic and predictive values for mortality and recurrence 9, 10, 11. However, the prognostic value of mixed histological predominant subtypes is inconsistent, so further studies are warranted.

Matrix metalloproteinases (MMPs) are a diverse group of molecular markers for prognosis and prediction of lung cancer. High level of tissue MMP9 expression was significantly correlated with poor survival of surgical patients 12. Our previous study also showed a high expression of MMP9 tended to enhance the metastasis of NSCLC 13. However, most literature reports focused merely upon the expression level of mRNA or protein. The proteinase activity of MMPs may be a better quantitative indicator for providing important insights into the therapeutic strategies of patients with early‐stage lung adenocarcinoma. Our study may explore the prognostic significance of MMP9 activity level and elucidate its correlation with NSCLC subtypes.

## Materials and Methods

### Cell lines and cell culture

Human lung adenocarcinoma cells SPC‐A‐1, SPC‐A1BM, and normal human bronchial epithelium cell BEAS‐2B were obtained from Cellular Institute of Chinese Academy of Science (Shanghai, China) 14. SPC‐A‐1 and SPC‐A1BM cells were cultured in Roswell Park Memorial Institute (RPMI) 1640 medium (Gibco) supplemented with 10% fetal bovine serum (FBS) (Bioind). And BEAS‐2B cell line was cultured in Dulbecco's modified Eagle's medium (DMEM, Gibco) containing 10% FBS. All growth media contained 100 *μ*g/L streptomycin and 100 U/L penicillin. The cells were maintained at 37°C in a humidified incubator with 95% air and 5% CO_2_. Cells were regularly certified as free of mycoplasma contamination. Cells were rinsed several times and placed into sterile phosphate‐buffered solution (PBS) immediately before implantation.

### Patients

From February 2013 to March 2016, a total of 104 consecutive patients of stage I B lung adenocarcinoma were recruited from Shanghai Chest Hospital. Tumor stage was determined by the seventh revision TNM classification of NSCLC. Collecting fresh frozen specimens of human lung adenocarcinoma was approved by our institutional review board. The inclusion criterion was a diagnosis of primary stage I B lung adenocarcinoma with complete lobectomy and systematic nodal dissection. The patients could receive the doublets including platinum plus a third‐generation agent (gemcitabine, vinorelbine, paclitaxel, pemetrexed) as adjuvant chemotherapy. And the exclusion criteria included incomplete resection or prior induction therapy. The relevant clinical data were retrieved from our institutional medical record system. The follow‐ups were conducted once every 3 months.

### Histological evaluation

All samples were fixed in 10% neutral‐buffered formalin, embedded in paraffin, and stained routinely with hematoxylin and eosin. For each case, the average number of reviewed slides was 10 (4–22). According to the IASLC/ATS/ERS classification scheme, the specimens were examined using comprehensive histological subtyping. The percentage was recorded in 5% increments for each histological component. Invasive adenocarcinomas were divided into five predominant groups of lepidic predominant (Lep), papillary predominant (Pap), acinar predominant (Aci), micropapillary predominant (MIP), and solid predominant (Sol). The predominant pattern was defined as the pattern with the greatest percentage.

### Transwell migration assay

Migration assay was performed with 24‐well microchemotaxis chamber (Costar) with uncoated polycarbonate membrane (pore size 8 *μ*m). Briefly, the cells were harvested and resuspended at 5 × 10^4^ cells/0.1 mL in DMEM containing 0.25% bovine serum albumin (BSA). Then, SPC‐A‐1, SPC‐A1BM, and BEAS‐2B cells were resuspended at 2 × 10^4^ cells/0.1 mL into the same media. Some bottom chambers of each Transwell unit contained 0.6 mL DMEM supplemented with 0.25% BSA and a chemically defined chemoattractant. In other bottom chambers, each Transwell unit contained 0.6 mL conditioned media from other cell cultures. The plates were incubated for 4 h at 37°C and the filters were fixed with saline‐buffered formalin and stained with 0.1% crystal violet solution. The cells migrating through the filter were counted with a grid under a microscope of 20× magnification.

### Assay of transient TIMP‐2 RNA interferences

Small interfering RNA (siRNA) technology was used for silencing the expression of target gene TIMP‐2. Approximately, 2 × 10^5^ cells/well were seeded in 6‐well plates and grown to a cell density of approximately 40%. Then, the medium was replaced with serum‐free RPMI 1640. A mixture of siRNA and Lipofectamine RNAiMAX (Invitrogen) was added. After 6 h incubation, the cells were cultured in RPMI 1640 medium supplemented with 10% FBS. RNA and protein were extracted after transfection. After transfecting with siRNA for 24 h, the cells were cultured for 24 h in serum‐free RPMI 1640 medium. Conditioned medium was collected and centrifuged for preserving supernatant. The siRNA sequences used were as follows:

### Control siRNA,

Forward: 5'‐GCUGCGAGUGCAAGAUCACTT‐3'.

Antisense: 5'‐GUGAUCUUGCACUCGCAGCTT‐3' (Ribobio);

TIMP‐2,

Forward: 5'‐GTG TGG GGT CTC GCT GGA‐3',

Reverse: 5'‐ GGG TGG TGC TCA GGG TGT C‐3';

### Immunohistochemical staining

Immunohistochemical (IHC) staining was used for detecting the protein expression in paraffin‐embedded tissues. The sections were immersed in sodium citrate repair solution (pH 6.0) under high pressure for 3 min, soaked in 3% H_2_O_2_ for 30 min, and then blocked with normal goat serum at 37°C for 1 h. Rabbit anti‐human MMP‐9 antibody was used for staining. After overnight incubation at 4°C, the slides were incubated with general immunohistochemical secondary antibodies (DAKO) for 30 min at 37°C and then visualized with DAB. The immunoreactivity scores of tissue samples were determined by the staining intensity and area of positive staining according to the method of Sun et al. Two independent pathologists evaluated all slides.

All sections were independently evaluated by two pathologists using a semiquantitative system based on the H‐index 15: 3 × percentage of strongly staining cells + 2 × percentage of moderately staining cells + percentage of weakly staining cells, giving “composite scores” that ranged from 0 to 300. All the cases were classified by the composite scores. Cases with the scores of 0–100 were interpreted as negative/mildly positive, 101–200 as moderately positive, and 201–300 as strongly positive. We divided 104 cases into MMP9 high‐expression (moderately/strongly positive) group and MMP9 low‐expression (negative/mildly positive) group.

### Assay of MMP enzyme activity

Fresh frozen cancer tissues were homogenized in 1x PBS by homogenizing machine (F6/10‐6G, FLUKO, Shanghai, China). After homogenization, the lysates were centrifuged for removing debris (5 min at 10,000*g*) and preserving supernatant. SPC‐A‐1, SPC‐A1BM, and BEAS‐2B cells were homogenized with 1xPBS by vortex. After homogenization, the lysates were centrifuged for removing debris (10 min at 13,000*g*) and preserving supernatant. The protein concentration of lysate was assayed by bicinchoninic acid (BCA) assay for MMP activity.

The tissue or cell MMP9 activity was measured by SensoLyte® 520 fluorimetric MMP assay kit (SensoLyte® Technology, Fremont, CA). And MMP9 activity was separately quantified. Briefly, 50 *μ*L culture medium was activated with 1 mmol/L APMA at 37°C for 3 h and then incubated in a 96‐well plate with the MMP‐9 FRET substrate for 60 min at 37°C. Fluorogenic substrate 5‐FAM/QXL520 was used in both kits. Upon cleavage by MMP9, fluorescence intensity was measured via ex/em = 480 + 20 nm/528 + 20 nm. The fluorescent signal was measured by VICTOR3^™^ Multilabel Counter (Perkin‐Elmer, Waltham, MA). And the concentration of MMP9 activity (ng/mg protein) was normalized by sample protein concentration.

### Statistical analysis

All data were analyzed by the SPSS13.0 statistical software (IBM SPSS, Chicago, IL, USA). Plotting and image processing were performed with Graphpad 6. A two‐tailed t‐test was used for examining the relationship between MMP9 activity and clinicopathological characteristics. The prognostic impact of each variable was evaluated by Kaplan–Meier method and log‐rank test during univariate survival analysis. Multivariate survival was analyzed with the Cox proportional hazard model for evaluating the independent prognostic factors for lung adenocarcinoma. *P *<* *0.05 was considered statistically significant and *P *<* *0.001 extremely statistically significant.

## Results

### Enzymatic activity of MMP9 and invasiveness in vitro

**Figure 1:**
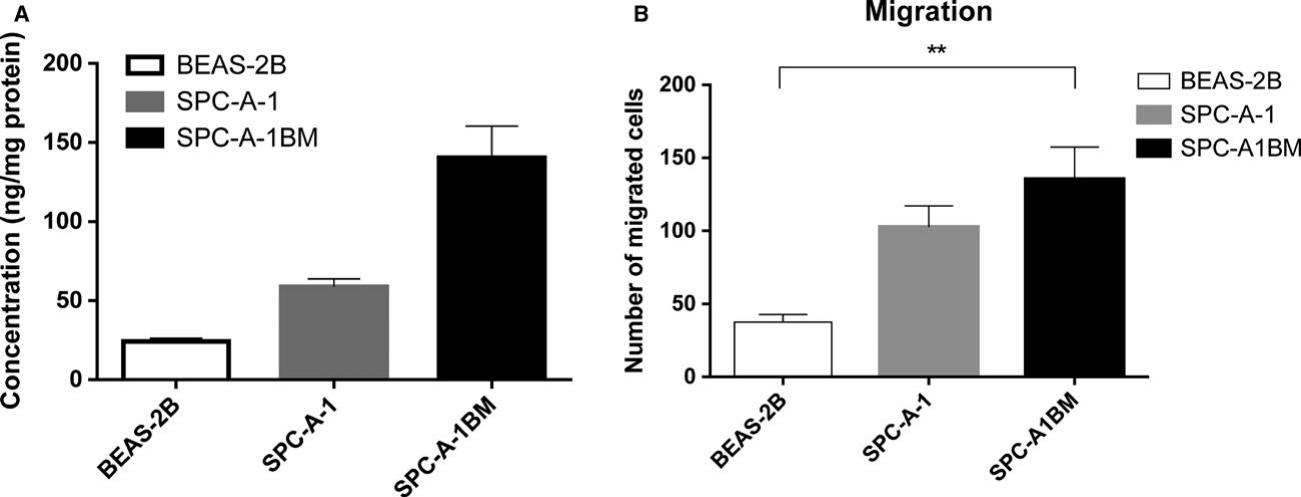
Matrix metalloproteinase 9 (MMP9) activity levels in *adenocarcinoma* cells and their invasive abilities (A) MMP9 activity levels determined with SensoLyte^®^ method; (B) Transwell filter assay validated invasive abilities of adenocarcinoma cells. **Significant P‐values (<0.001).

The activities of MMP9 were quantified. Significant differences existed between SPC‐A‐1 and SPC‐A‐1BM and control. SPC‐A‐1BM cell line demonstrated a potent capacity of bone metastasis in our previous work 14. It was more invasive than its parental cell line. And invasiveness was confirmed by Transwell migration assay. Thus, it became apparent that the more invasive cells, the higher levels of MMP9 activity (Fig. 1).

### Regulation of MMP9 activity via TIMP‐2 RNA interference in vitro

**Figure 2:**
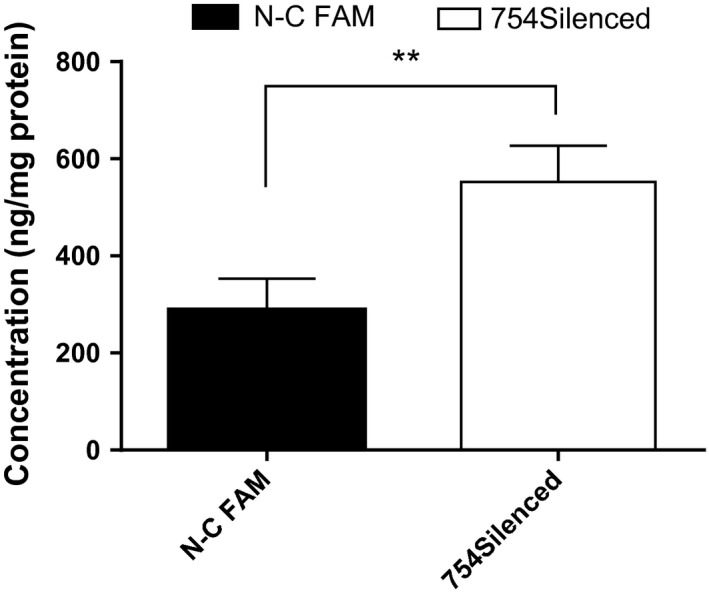
SensoLyte^®^ validation enzyme activity of MMP9 in SPC‐A‐1BM when TIMP2 inhibited. **Significant P‐values (<0.001).

As proved by the above tests in vitro, the MMP9 activity levels were correlated with adenocarcinoma invasiveness. Before replicating in clinical specimens, we attempted to know whether the MMP9 activity level was sufficiently sensitive for measurements. Tissue inhibitor of matrix metalloproteinase‐2 (TIMP‐2) is well known for its ability of regulating extracellular matrix degradation by inhibiting the proteolytic activities of MMPs 16, 17, 18. The results of RNA interference validated it. It was obvious that the MMP9 activity levels fluctuated significantly (*P *<* *0.001) when TIMP‐2 was suppressed (Fig. 2).

### Patient characteristics

For this study cohort, a total of 104 patients with stage I B invasive lung adenocarcinoma were recruited. The predominant subtypes were lepidic predominant subtype (*n* = 20), papillary predominant subtype (*n* = 22), acinar predominant subtype (*n* = 22), micropapillary predominant subtype (*n* = 20), and solid predominant subtype (*n* = 20). The median follow‐up period was 34 months (range: 24–37 months), and the last follow‐up time was March 2016; median age was 65 years (range: 46–81 years). Of the 104 patients identified, 37 patients experienced recurrence after surgery. Among them, there were 24 cases of distant recurrence and 13 cases of locoregional‐only recurrence. At the conclusion of study, there were 11 deaths. The 30‐month disease‐free survival (DFS) and overall survival (OS) were 64.2% and 88.9%, respectively. On the other hand, the 30‐month DFS of high and low expression of MMP9 were 63.6% and 65.0%. And the correlation between MMP9 expression and prognosis was statistically insignificant (*P *=* 0*.911). According to univariable analysis, lepidic predominant (*P *=* 0*.016), solid predominant (*P *=* 0*.007), and MMP9 activity level (*P *<* 0*.001) could predict postoperative recurrence (Table 1). Multivariate analysis revealed that pathological subtype and activity of MMP9 were independent prognostic factors for disease‐free survival, respectively (*P = *0.005 and 0.029) (Table 2).

**Table 1:** Patient characteristics

Characteristic	No. (%)	30 months DFS Rate, %	*P* a
Age, years			0.066
≤65	52 (50)	55.47	
>65	52 (50)	73.08	
Gender			0.397
Female	48 (46.2)	68.75	
Male	56 (53.8)	60.42	
Smoking history			0.063
Ever	49 (47.1)	54.66	
Never	55 (52.9)	72.73	
Size Grade			0.052
>3.0, ≤4.0 cm	74 (71.2)	71.60	
>4.0, ≤5.0 cm	30 (28.8)	52.50	
Acinar predominant			0.133
Yes	22 (21.2)	77.27	
No	82 (78.8)	60.75	
Lepidic predominant			**0.016**
Yes	20 (19.2)	84.00	
No	84 (80.8)	59.52	
Micropapillary predominant			0.066
Yes	20 (19.2)	45.00	
No	84 (80.8)	68.81	
Papillary predominant			0.133
Yes	22 (21.2)	77.27	
No	82 (78.8)	60.75	
Solid predominant			**0.007**
Yes	20 (19.2)	35.00	
No	84 (80.8)	71.20	
ECOG performance status			0.822
0	91 (87.5)	64.59	
1	13 (12.5)	61.54	
Visceral pleural involvement			0.589
Yes	33 (31.7)	60.10	
No	71 (68.3)	66.20	
Adjuvant chemotherapy			0.960
Yes	25 (24.0)	64.00	
No	79 (76.0)	64.26	
Lymphatic and/or vessel invasion			0.680
Yes	45 (43.3)	62.46	
No	59 (56.7)	66.67	
MMP9 activity level^b^			**<0.001**
Low	52 (50)	84.14	
High	52 (50)	44.23	
MMP9 expression level			
Low	49 (47.1)	65.03	0.861
High	55 (52.9)	63.64	

DFS, disease‐free survival.

a Significant *P*‐values (<0.05) are shown in **bold** type.

b For the sake of simplicity, we divided the different subtypes as high and low activity levels with the active MMP9 concentration in their median number.

**Table 2:** Multivariate analyses for disease‐free survival

Variables	HR	95% CI	*P* a
Age, years (≤65 vs. >65)	0.524	0.243–1.129	0.099
Gender (female vs. male)	0.163	0.013–2.043	0.160
Smoking history (never vs. ever)	11.760	0.937–147.551	0.056
Size Grade (>3.0, ≤4.0 cm vs. >4.0, ≤5.0 cm)	1.771	0.858–3.657	0.122
Predominant histologic subtypes (MIP and Sol vs. the rest)	3.119	1.415–6.877	**0.005**
ECOG performance status (0 vs. 1)	1.480	0.520–4.212	0.463
Visceral vascular invasion (yes vs. no)	1.451	0.668–3.152	0.347
Adjuvant chemotherapy (yes vs. no)	0.684	0.296–1.582	0.375
Lymphatic and/or vessel invasion (yes vs. no)	1.006	0.493–2.055	0.986
MMP9 activity level (low vs. high)	2.858	1.112–7.346	**0.029**
MMP9 expression level (low vs. high)	0.911	0.507–2.142	0.911

Invasive adenocarcinomas were divided into five groups: lepidic predominant (Lep), papillary predominant (Pap), acinar predominant (Aci), micropapillary predominant (MIP), and solid predominant (Sol).

a Significant *P*‐values (<0.05) are shown in **bold** type.

### Correlation between enzymatic activity of MMP9 and adenocarcinoma subtypes

**Figure 3:**
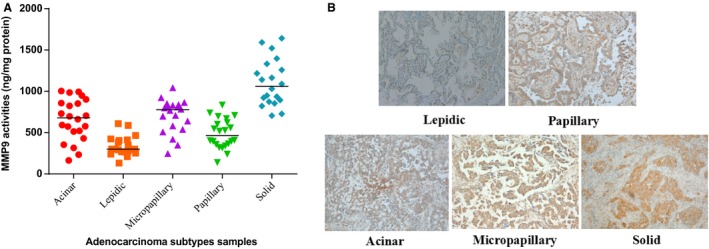
(A) SensoLyte^®^ validation enzyme activity level of MMP9 in the patients' tissues from different subtypes; (B) Immunohistochemical staining of MMP9 in five representative cases. Original magnification ×100. IHC scores of these five cases are lepidic, 86; papillary, 163; acinar, 182; micropapillary, 211; solid, 237. IHC, Immunohistochemical.

Based upon the measurements of enzymatic activity (Fig. 3A), there was a possible concentration gradient of active enzyme of MMP9 from low to high for lepidic predominant subtype, acinar predominant subtype, papillary predominant subtype, micropapillary predominant subtype, and solid predominant subtypes. The trend was significant (*P < *0.001 for lepidic predominance, micropapillary predominance, solid predominance, *P = *0.003 among acinar predominance and papillary predominance). From the results of immunohistochemical staining, the differential expressions of MMP9 were unclear for pathological subtypes (Fig. 3B, Table 1 and Table 2). Among these histological subtypes, 11 cases acinar predominant, 10 cases lepidic predominant, 10 cases micropapillary predominant, 11 cases papillary predominant, and 13 cases solid predominant patients were high expression of MMP9, and 11 cases acinar predominant, 10 case lepidic predominant, 10 cases micropapillary predominant, 11 cases papillary predominant, and seven cases solid predominant patients were low expression of MMP9. The differences between high and low expression MMP9 patients among different histological subtypes were insignificant (*P *=* 0*.937).

### Association between enzymatic activity of MMP9 and clinical outcomes of lung adenocarcinoma

**Figure 4:**
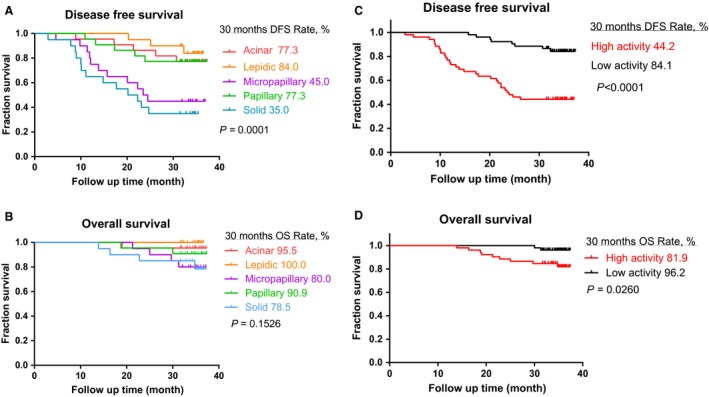
(A) Disease‐free survival (DFS) curve for the patients with stage I B invasive lung adenocarcinoma of five subtypes; (B) overall survival (OS) curve for the stage I B patients of five subtypes; (C) DFS curve for the patients with stage I B lung adenocarcinoma of MMP9 high and low activities; (D) OS curve for the stage I B patients of MMP9 high and low activities.

The relationship between MMP9 activity and clinical outcome of lung adenocarcinoma was significant. The active MMP9 concentrations were compared with their median concentrations. DFS was the lowest for solid predominant tumors, followed by micropapillary predominant tumors (Fig. 4A). However, the trend of OS was insignificant due to short follow‐up time (Fig. 4B). While analyzing DFS and OS by MMP9 activity levels, the patients with high‐level MMP9 activity tumors had significantly worse DFS and OS than those with low‐level MMP9 activity tumors (Fig. 4C and D). The 30‐month DFS rates were 44.2% and 84.1% (*P *<* *0.001); the 30‐month OS rates 81.9% and 96.2% (*P = *0.0260), respectively. In addition, the risk of recurrence peaked earlier for high‐level MMP9 activity tumors (6–12 months) than for low‐level MMP9 activity tumors (Fig. 4C). In addition, among the high‐level MMP9 activity patients, 27 cases and 25 cases were, respectively, with high expression and low expression of MMP9, and among the low‐level MMP9 activity patients, 28 cases and 24 cases were, respectively, with high expression and low expression of MMP9. The correlation between MMP9 expression and MMP9 activity was insignificant (*P *=* *0.84).

## Discussion

Our previous publications have shown that the 5‐year OS rate was disappointing at 60.87% for patients with stage I B NSCLC 19. Up to now, the role of adjuvant cisplatin‐based chemotherapy has been established by multiple large randomized phase III trials for resected stage II and IIIA NSCLC, but its role is controversial in stage I B patients. We need better prognostic factors to identify the patients with high risk of early recurrence, which may be helpful to select the patients suitable for adjuvant chemotherapy 20.

In our study, the 30‐month DFS rates of stage I B patients with lepidic predominant, acinar predominant, papillary predominant, micropapillary predominant, and solid predominant were 84.0%, 77.3%, 77.3%, 45.0%, 35.0%, respectively (*P *=* *0.0001). It further confirmed the relationship between histological subtypes of IALSC/ATS/ERS and prognosis. The new IASLC/ATS/ERS classification scheme of lung adenocarcinoma was proposed for identifying histological categories with prognostic differences. As reported by Yoshizawa et al., the IASLC/ATS/ERS classification could predict prognosis for stage I lung adenocarcinoma (*P *=* *0.038). The 5‐year DFS rates were 90.0%, 83.0%, and 84.0%, respectively, for lepidic predominant, papillary predominant, and acinar predominant subtypes. And the 5‐year DFS rates were 70.0% and 67.0% for solid predominant and micropapillary predominant subtypes 21. Our colleagues investigated 176 patients with stage IA lung adenocarcinoma undergoing lobectomy and mediastinal lymph node dissection. The patients with micropapillary predominant or solid predominant subtype had worse prognosis than other subtypes 22.

MMPs are a family of secreted or membrane‐associated metallic ion (usually zinc and sometimes cobalt) endopeptidases capable of digesting extracellular matrix components 23, 24. Especially MMP‐2 and MMP‐9 play vital roles in metastatic processes 25, 26. Expression of genes involved in tumor cell invasion and metastasis were upregulated between 10‐fold (MMP2) and 5000‐fold (MMP9), increased gene expression of MMP9, and decreased gene expression of tumor suppressor p53 was associated with poor tumor differentiation 27, 28. Moreover, the level of MMP9 expression was also correlated with cancer classification and overall survival of breast cancer 29, gastric cancer 30, prostate cancer 31, and NSCLC 32. In our study, MMP9 showed distinctive biological activity levels in different subtypes of lung adenocarcinoma. Thus, the significance of exploring the roles of MMP9 has been confirmed for tumor initiation and progression in some subtypes of IALSC/ATS/ERS lung adenocarcinoma.

Regarding biological functions, protein activity is more accurate than expression, at both transcriptional and protein levels. Enzymatic activity and protein levels of MMP‐2 and MMP‐9 were employed for evaluating the migratory and invasive capacities of NSCLC cell lines A549 33. To the best of our knowledge, the prognostic value of MMP9 activity has not been examined for invasive lung adenocarcinoma resection specimens. Moreover, the relationship between qualitative factors and new classification system has remained unknown.

Here, a validated method was employed to determine the activity of MMP9 in human lung adenocarcinoma cells. As seen from the results in vitro, the determined MMP9 activities of cell lines reflected cellular invasiveness. The SensoLyte® assay was proven to be sensitive for TIMP‐2 silencing MMP9 activities in NSCLC cell lines. Thus, this method could determine the MMP9 activity of fresh frozen specimens from five lung adenocarcinoma subtypes. Significant relationship existed between MMP9 activity level and adenocarcinoma subtypes. Based on the follow‐up data, there was significant relationship between enzymatic activity of MMP9 and prognosis.

Some limitations existed in experimental designs. The sample size of patients' tissue was small and the follow‐up time was insufficient for overall survival study. However, MMP9 activity measurement has great prognostic values for early‐stage lung adenocarcinoma patients. Our results also showed that the MMP9 activity was the highest in solid predominant and micropapillary predominant subtypes, intermediate in acinar predominant and papillary predominant subtypes, and the lowest in lepidic predominant subtype. And such a tendency was in accordance with subtypic prognoses.

In summary, pathological subtype and MMP9 activity level are independent prognostic factors. A high‐level MMP9 activity is correlated with aggressive tumor behaviors and poor clinical outcomes in early‐stage lung adenocarcinoma after complete resection.

## Ethical Approval

All procedures performed in studies involving human participants were in accordance with the ethical standards of the institutional and/or national research committee and with the 1964 Helsinki declaration and its later amendments or comparable ethical standards.

## Informed Consent

Informed consent was obtained from all individual participants included in the study.

## Conflict of Interest

The authors have no conflicts of interest.

## Supporting information


**Data S1**. Original Data Set.
